# Evaluation of Major Online Diabetes Risk Calculators and Computerized Predictive Models

**DOI:** 10.1371/journal.pone.0142827

**Published:** 2015-11-11

**Authors:** Gregor Stiglic, Majda Pajnkihar

**Affiliations:** 1 Faculty of Health Sciences, University of Maribor, Maribor, Slovenia; 2 Faculty of Electrical Engineering and Computer Science, University of Maribor, Maribor, Slovenia; University of Rochester, UNITED STATES

## Abstract

Classical paper-and-pencil based risk assessment questionnaires are often accompanied by the online versions of the questionnaire to reach a wider population. This study focuses on the loss, especially in risk estimation performance, that can be inflicted by direct transformation from the paper to online versions of risk estimation calculators by ignoring the possibilities of more complex and accurate calculations that can be performed using the online calculators. We empirically compare the risk estimation performance between four major diabetes risk calculators and two, more advanced, predictive models. National Health and Nutrition Examination Survey (NHANES) data from 1999–2012 was used to evaluate the performance of detecting diabetes and pre-diabetes.

American Diabetes Association risk test achieved the best predictive performance in category of classical paper-and-pencil based tests with an Area Under the ROC Curve (AUC) of 0.699 for undiagnosed diabetes (0.662 for pre-diabetes) and 47% (47% for pre-diabetes) persons selected for screening. Our results demonstrate a significant difference in performance with additional benefits for a lower number of persons selected for screening when statistical methods are used. The best AUC overall was obtained in diabetes risk prediction using logistic regression with AUC of 0.775 (0.734) and an average 34% (48%) persons selected for screening. However, generalized boosted regression models might be a better option from the economical point of view as the number of selected persons for screening of 30% (47%) lies significantly lower for diabetes risk assessment in comparison to logistic regression (p < 0.001), with a significantly higher AUC (p < 0.001) of 0.774 (0.740) for the pre-diabetes group.

Our results demonstrate a serious lack of predictive performance in four major online diabetes risk calculators. Therefore, one should take great care and consider optimizing the online versions of questionnaires that were primarily developed as classical paper questionnaires.

## Introduction

Early identification of persons at increased risk of developing type 2 diabetes is of high importance. One of the common approaches to effect changes in lifestyle is screening of populations to detect persons at risk using self-assessment questionnaires.

Studies comparing paper to electronic questionnaires date back over a decade, with most of them focusing on user experience and perception of the electronic questionnaires in comparison to their paper-and-pencil versions. Cook et al. [1] compared electronic to paper questionnaires for chronic pain assessment in a randomized, crossover study. Their results support the validity and acceptance of electronic versions with the majority of users rating e-questionnaires as easier to use and preferable.

Today, most national diabetes associations publish their population diabetes screening questionnaires in paper-and-pencil as well as online forms. However, most online screening tools are directly copied from the paper versions to the online environment. Therefore, one can notice that there are very few diabetes predictive models that are not based on multiple logistic regression or very similar techniques that can be easily transformed into a simple scoring system where the user only has to add up the points scored for each answer in the questionnaire.

A study by Yu et al., [2] proposed a novel diabetes and pre-diabetes screening approach using Support Vector Machine (SVM) techniques. Although their results show that, they can outperform classical techniques in terms of Area Under the ROC Curve (AUC), it is only available as a web application that was developed as a demonstration and supplement to the published paper available.

The problem addressed in this present study is not only the use of logistic regression in most screening tools published by major diabetes associations, but the fact that such models are not used directly, i.e. without transformation to integer-based scoring system for risk estimation in the online environment. A recent study by Collins [3] points out that approximately 57% of the prediction models in the literature are presented and evaluated as simplified scoring systems. They emphasized that it was often unclear whether the reported performance measures were for the full regression model or for the simplified models. It has to be noted that there are also positive sides of using a simplified scoring systems, such as ease of use for general practitioners and patients themselves, but only when they are used in the classical paper form.

This study compares four widely used diabetes and pre-diabetes screening tools and a logistic regression predictive model used directly to predict the risk of diabetes and pre-diabetes. Additionally, we observe the performance of a more complex boosting based predictive model to demonstrate the loss in performance that can be inflicted when integer-based scoring systems are used in an online environment. A comparison of predictive performance (AUC) of six methods in this paper is supplemented by direct comparison of agreement between three best performing models. Model output agreement analysis allows identification of input variables and their specific values that can help us explain why computerized predictive models deployed online outperform more traditional paper-and-pencil versions of screening tests.

## Methods

### Modelling Online Risk Calculators

Although there are multiple diabetes risk prediction models available [4, 5], we used four models in this study based on potential outreach in the general population. We chose four online diabetes risk calculators from national diabetes associations in the US, UK, Canada and Australia, assuming that national diabetes association websites attract the highest number of potential users for diabetes risk calculators. To compare performance of the observed calculators, we built a model for each online questionnaire. Table 1 presents a list of variables that were mapped from questions in the questionnaires to the NHANES variables. As we had to adapt some of the models due to limited availability of data for three variables, we used a threshold tuning process for all four models. It has to be noted that the purpose of Leicester Risk Assessment (LRA) and Australian Type 2 Diabetes Risk Assessment Tool (AUSDRISK) calculators differ from American Diabetes Association Questionnaire (ADA) and Canadian Diabetes Risk Questionnaire (CANRISK) calculators in the fact that they were originally developed to estimate the risk of diabetes in the future instead of detecting undiagnosed diabetes or pre-diabetes. However, by tuning the threshold score parameter on the training set, we optimized the output score from the LRA and AUSDRISK calculators to predict the current risk of type 2 diabetes. This step involved the training data that was used to train the classification models. However, in case of four questionnaire-based models, we used the training set to find the optimal threshold that would maximize the AUC. By setting the optimal thresholds using training data, we could test each questionnaire in both scenarios to identify persons at risk of diabetes as well as prediabetes. Once the threshold for the model was set, we were able to test the model on test data. The threshold was set for each holdout evaluation separately to avoid bias in performance estimation [6]. Threshold candidate values were taken from intervals of integer values ranging from minimal to maximal possible score for ADA, LRA and AUSDRISK. In case of eCANRISK, where outcome is not an integer, we tested for optimal threshold value by increasing the threshold in steps of 0.1.

**Table 1:** Presence of questions in four compared diabetes online risk calculators with corresponding score intervals used in this study. Variables in bold represent a set of common variables that are used in the majority of compared online calculators.

Question	ADA	eCANRISK	LRA	AUSDRISK
**Age**	+	+	+	+
**Sex**	+	+	+	+
**Diabetes in family**	+	+	+	+
**High blood pressure history**	+	+	+	
Blood pressure medication history		+	+	
Taking blood pressure medication				+
**Physical activity**	+	+		+
**Obesity (BMI)**	+	+	+	
Gestational diabetes^1^	+	+		
**Waist measurement**		+	+	+
Eats vegetables and fruit^1^		+		+
High blood sugar history^2^		+		+
Ethnic group/Country of birth		+	+	+^1^
Level of education		+		
Smoking				+
Score interval	0–10	0–87	0–46	0–27

^1^Not used in the experiments due to limited availability in NHANES 1999–2012.

^2^Used to remove individuals with already diagnosed diabetes.

The following subsections outline the main characteristics of original questionnaires, their online versions and studies providing more information on development of the underlying models as summarized in Table 2.

**Table 2:** Summary of questionnaires used in this study. Comparison is provided for maximal number of points a user can score, cutpoint value for high risk and number of questions in the questionnaire. Additionally, datasets used for development and validation of the model are provided along with the corresponding reported predictive performance values.

Questionnaire	Max points	Cutpoint	Num. questions	Datasets used	Reported predictive performance (AUC)
**ADA [9]**	11	≥ 5	7	Development: NHANES 1999–2004 Validation: NHANES 2005–2006, ARIC and CHS	0.83 (NHANES) 0.72–0.74 (ARIC/CHS)
**CANRISK [17]**	86	≥ 21 MR ≥ 33 HR	13	CANRISK study (6223 participants)	0.75
**LRA [18]**	47	≥ 14	8	LRA study (6390 participants)	0.72
**AUSDRISK [24]**	35	≥ 12		AusDiab 1999–2005 (6060 participants) Validation: BMES (1993 participants), NWAHS (1465 participants)	0.78

MR–moderate risk, HR–high risk

#### American Diabetes Association Questionnaire (ADA)

This widely used online calculator is available from the official ADA website [7] and is based on the slightly adapted methodology that was published in a study by Bang et al. [8]. The same online version of the questionnaire is also available at the National Diabetes Education Program (NDEP) website [9] hosted at the National Institutes of Health (NIH). In comparison to the original study, the online version also includes a question on gestational diabetes. The threshold for this questionnaire is set at 5 points (out of 11 possible), instructing all users who scored above this threshold that they are at increased risk for having type 2 diabetes. Users at risk are further advised to see their doctor and check if additional testing is needed. In comparison to other calculators included in this study, ADA asks only seven questions and is the simplest in terms of effort needed to fill in the questionnaire. The ADA model was developed using NHANES data 1999–2004 and validated on NHANES 2005–2006, Atheroscleriosis Risk in Communities (ARIC) [10] and Cardiovascular Health Study (CHS) [11] studies. The final model by Bang et al. yielded an AUC of 0.83 on NHANES, 0.74 on ARIC/CHS for diabetes risk estimation and 0.72 for pre-diabetes.

#### Canadian Diabetes Risk Questionnaire (CANRISK)

The online version of the CANRISK calculator is hosted at the Governement of Canada website [12], with an additional link from the Canadian Diabetes Association [13]. With 13 questions, CANRISK represents the most complex questionnaire and it takes the most effort for a user to answer all questions. In contrast to ADA, CANRISK uses two threshold values and stratifies persons into three categories of having pre-diabetes or type 2 diabetes: low risk (cumulative score of less than 21), moderate risk (21–32) and high risk (33 and over) with maximal score of 86. In case of moderate risk, users are advised to consult with health care practitioner about their risk of developing diabetes. For the high-risk group, the questionnaire suggests to consult with a health care practitioner to discuss getting blood sugar tested. CANRISK is based on the Finish Diabetes Risk model (FINDRISK) with adaptations to reflect Canada’s multi-ethnic population [14]. CANRISK is the only calculator where we were able to find a study that provided an electronic format of the score calculation called eCANRISK. This is the only case where it was explicitly advised to use a different approach to score computation when developing an online or mobile version of the application. The CANRISK validation study by Robinson et al. provides a regression model coefficients that can be used for “programmed risk calculators (e.g. iPad App, online web calculator)” and an additional paper-based format [15]. It is important to note that Robinson et al. validated CANRISK and eCANRISK on 6223 adults of various ethnicities and obtained the same AUC scores for both versions (0.75, 95% CI: 0.73–0.78). In this study we use the eCANRISK score based on regression coefficients that is also used for the online CANRISK calculator.

Leicester Risk Assessment (LRA). The Leicester Risk Assessment score is an impaired glucose regulation and type 2 diabetes mellitus risk assessment tool developed by Gray et al. [16] for the multiethnic UK population. The online version of this questionnaire is available at Diabetes UK [17] and UK National Health Service (NHS) [18] websites. Data from 6390 participants aged 40–75 was used to develop the risk assessment tool. External validation of the score was conducted using data from 3171 participants from a separate screening study and yielded an AUC of 0.72 (95% CI: 0.69–0.74). The questionnaire includes eight questions (Table 1) with the score range of [0–47], where a higher score corresponds to higher risk.

#### Australian Type 2 Diabetes Risk Assessment Tool (AUSDRISK)

In comparison to the first three online risk assessment tools that focused on identification of persons with high risk of diabetes or pre-diabetes, AUSDRISK focuses on assessment of risk of developing type 2 diabetes over the next five years. The online version of the questionnaire is available from The Australian Department of Health website [19] with additional link from the Diabetes Australia website [20]. Data from AusDiab-Australian Diabetes, Obesity and Lifestyle study (1999–2000) [21] with a five-year follow up (2004–2005) was used to develop this risk assessment tool [22]. In the five-year period 362 people out of 6060 from the AusDiab study developed diabetes. Data from 1993 participants of the Blue Mountains Eye Study (BMES) [23] and 1465 participants from the North West Adelaide Health Study (NWAHS) [24] was used to validate the risk assessment tool. The AUC of AUSDRISK was 0.78 (95% CI: 0.76–0.81) using a score threshold of 12 out of the maximal 35 points.

Mapping Limitations. Table 3 summarizes the mapping process to define which NHANES variable can map to a specific question in a risk assessment test and in some cases also solve the problem of different questions in different NHANES waves.

In the ADA questionnaire there was a problem with mapping a gestational diabetes question, because this question exists only in the two most recent waves (2009–2010 and 2011–2012) of NHANES. Including this variables would introduce a large number of missing values (more than 70%), therefore this variable was omitted from this analysis (the value was always zero).

**Table 3:** Overview of variables and their mappings to variables and questions from seven waves of 1999–2012 NHANES data.

Question / Variable	NHANES Variable	NHANES Questions with additional notes
Age	RIDAGEYR	Age in years (capped at 85)
Sex	RIAGENDR	Gender (Male, Female)
Diabetes in family	MCQ260Ax (Waves 1–3)	Which biological family member? (Mother, Father, Grandmother, Grandfather, Brother, Sister, Other family member)
	MCQ300C(Waves 4–7)	Including living and deceased, were any of your close biological (blood) relatives including father, mother, sisters or brothers, ever told by a health professional that they had diabetes?
High blood pressure history	BPQ020	Have you ever been told by a doctor or other health professional that you had hypertension, also called high blood pressure?
Blood pressure medication history	BPQ040A	Because of your (high blood pressure/hypertension), have you ever been told to take prescribed medicine?
Taking blood pressure medication	BPQ050A	Are you now taking prescribed medicine?
Physical activity	PAD200 (Waves 1–4)	Over the past 30 days, did you do any vigorous activities for at least 10 minutes that caused heavy sweating, or large increases in breathing or heart rate? Some examples are running, lap swimming, aerobics classes or fast bicycling.
	PAQ650 (Waves 5–7)	Do you do any vigorous-intensity sports, fitness, or recreational activities that cause large increases in breathing or heart rate, like running or basketball for at least 10 minutes continuously?
Obesity / BMI	BMXBMI	Body Mass Index (kg/m^2^)
Gestational diabetes^1^	RHQ162 (Waves 6–7)	During any pregnancy, were you ever told by a doctor or other health professional that you had diabetes, sugar diabetes or gestational diabetes? Please do not include diabetes that you may have known about before the pregnancy. Note: only available in NHANES 2009–2010 and 2011–2012!
Waist measurement	BMXWAIST	Waist circumference in cm.
Eat vegetables and fruit^1^	FFQ0016-19 FFQ0022 FFQ0032-34 FFQ0036 FFQ0039 (Waves 3–4)	How often did you eat fruit {apples, pears, bananas, pineapples, grapes}? AND How often did you eat vegetables {carrots, string beans, peas, broccoli, onions}? Note: only available in waves 3 and 4 of NHANES data, therefore we did not include this variable in the final set of variables for this study.
Ethnic group / Country of birth	RIDRETH1	Race/Ethnicity (Mexican American, Other Hispanic, Non-Hispanic White, Non-Hispanic Black and Other Race)
Level of education	DMDEDUC2	What is the highest grade or level of school you have completed? (Less than 9th Grade, 9–11th Grade, High School Grad/GED or Equivalent, Some College or AA degree, College Graduate or above)
Smoking	SMQ040	Do you now smoke cigarettes? (Every day, Some days, Not at all) Note: everything above “Not at all” was treated as a positive answer.

^1^Not used in the experiments due to limited availability in NHANES 1999–2012

Questions asking about gestational diabetes and eating vegetables and fruit were also omitted from our model for the CANRISK questionnaire. A study by Robinson et al. [15] on validation of CANRISK presented results where gestational diabetes represents a variable with the lowest β-coefficient in the regression model. Although we were able to identify questions (Table 3) in NHANES waves 3 and 4 that would allow us to check if specific fruits and vegetables were consumed by participants, we would again face a large number of missing values, therefore this variable that was also excluded in this study. CANRISK and AUSDRISK also include a question on blood sugar history that was used to define a class of undiagnosed diabetes and is therefore not used in this study.

We were able to define mappings for all questions in the LRA questionnaire, but a question on ethnicity and country of birth in AUSDRISK should be highlighted. This question in AUSDRISK questionnaire is divided into two parts: (a) Are you of Aboriginal, Torres Strait Islander, Pacific Islander or Maori descent? and (b) Where were you born? The user would score two points for answering yes to the first question and an additional two points if born in Asia, Middle East, North Africa, or Southern Europe. As we do not have this kind of information in NHANES, we did not use this variable for AUSDRISK models. It has to be noted that this variable influences the final result only for a small percentage of people even in Australian population.

### Datasets

The experimental work in this paper is based on seven cross-sectional waves of NHANES data from 1999–2000 and 2011–2012 [25]. Only persons over 20 years of age, excluding pregnant women, were included in this study. A separate dataset derived from NHANES data were prepared for diabetes and pre-diabetes risk estimation. A fasting plasma glucose level ≥ 126 mg/dL was used to define a diabetes group of persons, while levels ≥ 100 mg/dL were used to define a positive group in the second (pre-diabetes) dataset. Persons from a positive group in both datasets who answered “yes” to the question “Other than during pregnancy, have you ever been told by a doctor or health professional that you have diabetes or sugar diabetes?” or taking insulin or oral medications for diabetes were removed from datasets. Altogether, there were 14207 persons with available data on fasting plasma glucose measurements. Diabetes dataset included 621 (4.4%) positive cases, while the pre-diabetes dataset included 5720 (40.3%) positive cases.

There were eight variables with missing values with up to 76% for variable representing whether a person takes any blood pressure medication, followed by questions on blood pressure medication history and smoking with 70% and 53% of missing values respectively. In presence of missing values, the value was set to zero with an additional dummy variable denoting the presence of missing values added to the dataset.

### Statistical Analysis

In addition to the simple scoring models of four major online diabetes risk calculators, this study empirically evaluated two additional approaches to risk assessment–i.e. logistic regression using Generalized Linear Models (GLM) and regression trees using Generalized Boosted Models (GBM), both implemented in R statistical language. GLM are a widely used family of regression models that allow non-gaussian distributions. Nelder and Winterburn describe it as a generalization that includes linear, logistic and Poisson regression [26]. In our case, a binary distributed outcome variable was used. The implementation of GBM implemented by Ridgeway [27] that was used in this study closely follows the Gradient Boosting Machine approach by Friedman [28]. Similar to GLM, GBM can be used for regression as well as classification problems. Friedman uses additive boosting to build multiple regression trees and even provides different methods for interpretation of results, which is an extremely important characteristic of a predictive model, especially in biomedical domain [29]. Default parameter values for GBM were used, except in case of interaction depth of decision trees where up to 3-way interactions were allowed and in numbers of iterations that were increased from 100 to 200 to obtain better performance with manageable computational complexity. Logistic regression models are the most widely used approached to building risk models and therefore GLM was used in this study as well. The motivation for choosing GBM as a second option to build a predictive model lies in the fact that it uses a concept of boosting the classifiers and is therefore conceptually very different from the GLM. It is notable that although GBM will outperform GLM in most cases, GLM are simple to interpret, while interpretation of results from GBM represents a challenging problem.

A stratified random sampling-based holdout evaluation, with 50% samples selected in training set and the remaining 50% in testing set, was used in all experiments. The holdout evaluation was repeated 1000 times to allow empirical estimation of confidence intervals for all performance metrics.

Six performance metrics were used to observe the classification performance of six compared diabetes risk calculation approaches. Initial comparisons of models were done using the area under the receiver operating characteristic curve (AUC), followed by sensitivity, specificity, positive predictive value (PPV) and negative predictive value (NPV). All six performance metrics were calculated on testing set in each of the 1000 experimental runs. The results were then visualized using box-plots that allow visualization of the performance metrics distribution, which is important when comparing different methods. Additionally, we used paired samples t-test with Bonferroni adjustment of p-values for multiple testing to estimate the significance of difference between the AUC values.

When comparing the agreement between specific risk calculators, Cohen’s Kappa [30] coefficient was calculated. Cohen’s Kappa can range from -1 for completely opposite outcomes to +1 representing a perfect agreement between the two compared vectors of outcomes. In contrast to the percentage of cases where two classifiers agree, Kappa takes into account the possibility that two classifiers agree simply by chance. Landis and Koch [31] define the Kappa values lower than 0 as no agreement, 0–0.20 as slight, 0.21–0.40 as fair, 0.41–0.60 as moderate, 0.61–0.80 as substantial, and 0.81–1 as almost perfect agreement.

All statistical analyses were performed using the R statistical language version 3.0.2 [32].

## Results

This section presents the results of empirical comparison, measuring the classification performance of four major online diabetes risk calculators, logistic regression and an alternative boosting-based approach. All experiments were conducted on two different datasets (diabetes and pre-diabetes) with two different sets of variables. The first experiment used all available variables that could be mapped from questions asked in the online risk assessment calculators to NHANES data, while the second experiment included only a set of variables that were common to all four online calculators. A full and common (in bold) sets of variables can be found in Table 1.

### Full Set of Variables

**Fig 1:**
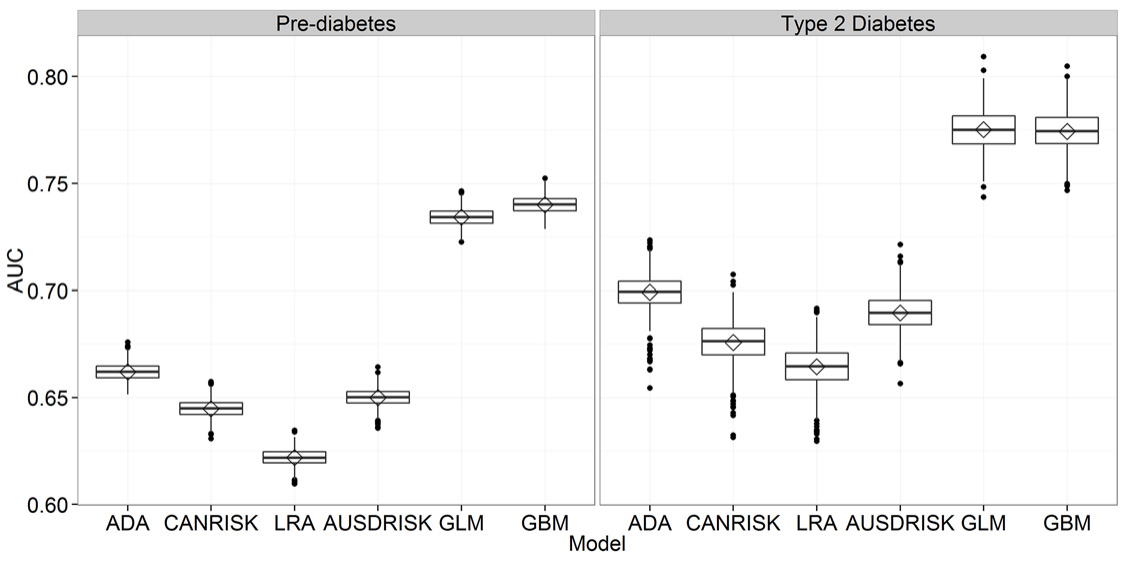
Comparison of AUC for six risk estimation approaches using all available variables.

In the experiments using full set of variables we used different number of variables for different predictive models ranging from six variables in the ADA model to a full set of 12 variables used with GLM and GBM models. Fig 1 presents AUC results for all six predictive models, where it is possible to observe the significant gap in performance between the questionnaire based models and both proposed predictive models. As expected, the two models where all available variables were used outperformed other models as seen from Fig 1. Comparing results of the diabetes vs. pre-diabetes one can notice higher variability of results in diabetes risk estimation originating from large class imbalance present in the diabetes risk estimation problem.

Table 4 represents additional performance metrics that allow comparison of predictive models from different perspectives. In pre-diabetes classification we can observe well balanced sensitivity and specificity values, especially for ADA, GLM and GBM, with the highest sensitivity at AUSDRISK and the highest specificity at GBM.

**Table 4:** Mean Specificity, Sensitivity, PPV, NPV and Percentage of selected persons (PSP) for 1000 holdout evaluations with corresponding 95% confidence intervals for all available variables.

Model	Sensitivity	Specificity	PPV	NPV	Percentage Selected
Pre-diabetes					
ADA	.667 [.655, .679]	.657 [.647, .667]	.567 [.559, .575]	.745 [.738, .753]	.474 [.466, .481]
eCANRISK	.660 [.595, .756]	.630 [.530, .691]	.547 [.519, .568]	.734 [.715, .764]	.487 [.424, .585]
LRA	.690 [.582, .781]	.554 [.462, .655]	.512 [.492, .533]	.728 [.698, .762]	.544 [.440, .635]
AUSDRISK	**.710 [.595, .745]**	.590 [.558, .693]	.539 [.528, .569]	.752 [.717, .768]	.531 [.423, .562]
GLM	.687 [.648, .719]	.661 [.633, .693]	.578 [.566, .591]	.758 [.744, .772]	.479 [.445, .508]
GBM	.685 [.653, .717]	**.671 [.640, .699]**	**.584 [.571, .597]**	**.760 [.747, .772]**	**.472 [.444, .503]**
Type 2 Diabetes					
ADA	**.852 [.826, .884]**	.546 [.536, .553]	.079 [.076, .082]	**.988 [.986, .990]**	.471 [.464, .481]
eCANRISK	.756 [.639, .819]	.596 [.548, .682]	.079 [.074, .085]	.982 [.977, .986]	.420 [.333, .467]
LRA	.769 [.684, .829]	.559 [.530, .619]	.074 [.070, .079]	.982 [.977, .986]	.455 [.395, .484]
AUSDRISK	.748 [.642, .874]	.631 [.518, .715]	.086 [.074, .098]	.982 [.977, .989]	.386 [.302, .498]
GLM	.727 [.639, .803]	.677 [.635, .727]	.093 [.088, .099]	.982 [.978, .986]	.340 [.289, .383]
GBM	.668 [.584, .745]	**.721 [.681, .762]**	**.099 [.092, .106]**	.979 [.976, .984]	**.296 [.254, .337]**

The strength of the GBM approach can be clearly seen when observing PPV, NPV and the percentage of selected cases, especially in pre-diabetes risk estimation problem. With the lowest percentage of selected cases (29,6%), GBM obtained the highest percentage of correctly classified cases in the group of persons selected for screening (58,4%). A very similar distribution of results can be observed in the diabetes risk estimation. It is important to note that low percentage of selected cases with high PPV results in cost reduction as less people need further, more expensive testing for diabetes.

### Minimal Set of Variables

**Fig 2:**
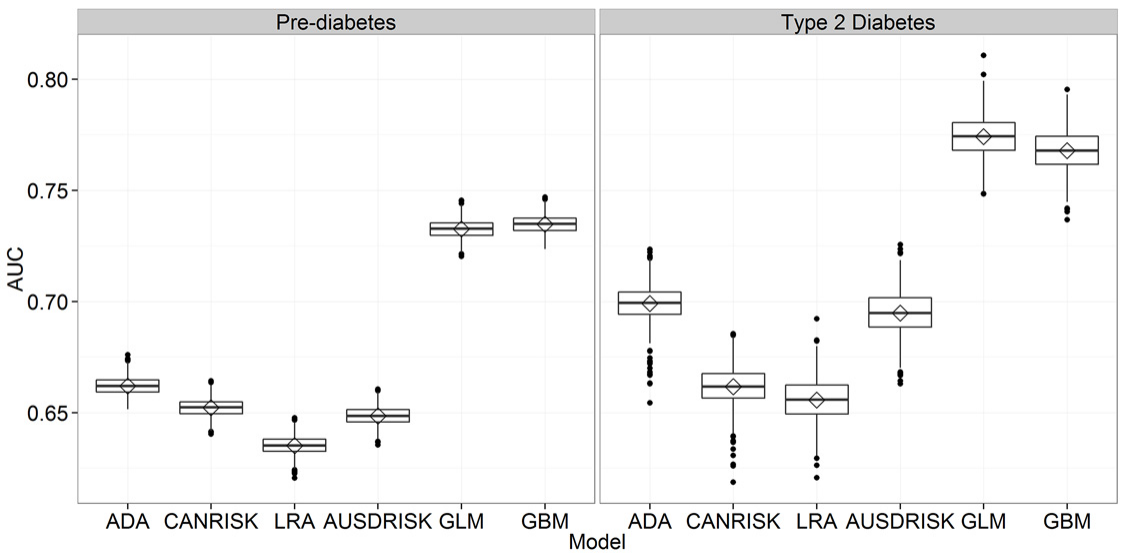
Comparison of AUC for six risk estimation approaches using a set of common variables.

To allow fair comparison, we identified a set of common variables that are used by at least three out of four compared questionnaire based models. In this way we obtained a minimal set of features including age, sex, diabetes family history, hypertension, physical activity, BMI and waist circumference. Compared to results using the full set of variables, there are no major differences (Table 5). Here, GLM performs slightly better in AUC for diabetes prediction (Fig 2), while GBM selects the smallest number of persons for screening. This is especially important in the diabetes prediction where even 29,7% of persons selected for screening is a large number, taking into account that the prevalence of diabetes in the NHANES 1999–2012 data lies at 4,4%. No specific techniques such as oversampling or undersampling were used to deal with class imbalance. This was done intentionally to allow for fair comparison to questionnaire based models where only the threshold can be adapted on the training set.

**Table 5:** Mean Specificity, Sensitivity, PPV, NPV and Percentage of selected persons (PSP) for 1000 holdout evaluations with corresponding 95% confidence intervals for a set of common variables.

Model	Sensitivity	Specificity	PPV	NPV	Percentage Selected
Pre-diabetes					
ADA	.667 [.655, .679]	.657 [.647, .667]	.567 [.559, .575]	.745 [.738, .753]	.474 [.466, .481]
eCANRISK	**.691 [.631, .736]**	.614 [.571, .671]	.547 [.532, .566]	.747 [.728, .766]	.509 [.452, .550]
LRA	.640 [.625, .681]	.630 [.576, .643]	.539 [.521, .548]	.722 [.714, .731]	.479 [.469, .529]
AUSDRISK	.661 [.549, .750]	.636 [.544, .742]	.552 [.524, .592]	.737 [.708, .764]	.484 [.377, .574]
GLM	**.691 [.658, .718]**	.657 [.631, .683]	.576 [.565, .587]	**.760 [.747, .772]**	.483 [.454, .509]
GBM	.673 [.639, .706]	**.674 [.642, .704]**	**.582 [.569, .597]**	.754 [.741, .766]	**.466 [.435, .498]**
Type 2 Diabetes					
ADA	**.852 [.826, .884]**	.546 [.536, .553]	.079 [.076, .082]	**.988 [.986, .990]**	.471 [.464, .481]
eCANRISK	.710 [.561, .871]	.613 [.454, .737]	.079 [.067, .094]	.979 [.973, .988]	.401 [.277, .561]
LRA	.700 [.529, .816]	.612 [.483, .765]	.077 [.067, .092]	.978 [.972, .984]	.402 [.247, .530]
AUSDRISK	.760 [.645, .865]	.630 [.509, .721]	.086 [.074, .094]	.983 [.978, .988]	.387 [.295, .507]
GLM	.736 [.648, .806]	.679 [.637, .722]	.095 [.089, .101]	.983 [.978, .987]	.339 [.296, .381]
GBM	.666 [.587, .739]	**.719 [.678, .763]**	**.098 [.091, .106]**	.979 [.975, .983]	**.297 [.253, .339]**

Comparison of AUC between the full and minimal set shows decrease of performance in some cases: GBM from 0.740 (95% CI: 0.732–0.747) to 0.735 (95% CI: 0.727–0.742) for pre-diabetes and from 0.774 (95% CI: 0.755–0.791) to 0.768 (95% CI: 0.750–0.785) for diabetes, CANRISK from 0.676 (95% CI: 0.654–0.694) to 0.662 (95% CI: 0.644–0.677) and LRA from 0.664 (95% CI: 0.645–0.683) to 0.656 (95% CI: 0.636–0.674), both for diabetes. However, a positive impact of feature selection can also be noticed in some cases where performance improved with the reduced set of variables. This was the case in pre-diabetes classification for LRA where AUC increased from 0.622 (95% CI: 0.614–0.629) to 0.635 (0.627–0.643) and CANRISK with an increase from 0.645 (95% CI: 0.637–0.653) to 0.652 (95% CI: 0.644–0.660).

### Agreement of Predictive Models

To obtain a better insight into the characteristics of specific models, a further analysis was conducted by comparing the agreement between the outputs of ADA and the two proposed predictive models. This experiment aimed to show in which cases the outcomes of simpler models differ from the proposed GLM and GBM approach. Consequently, the reasons for better performance of the GLM and GBM. Kappa statistics was used to measure the agreement between the outcomes of ADA, GLM and GBM.

**Fig 3:**
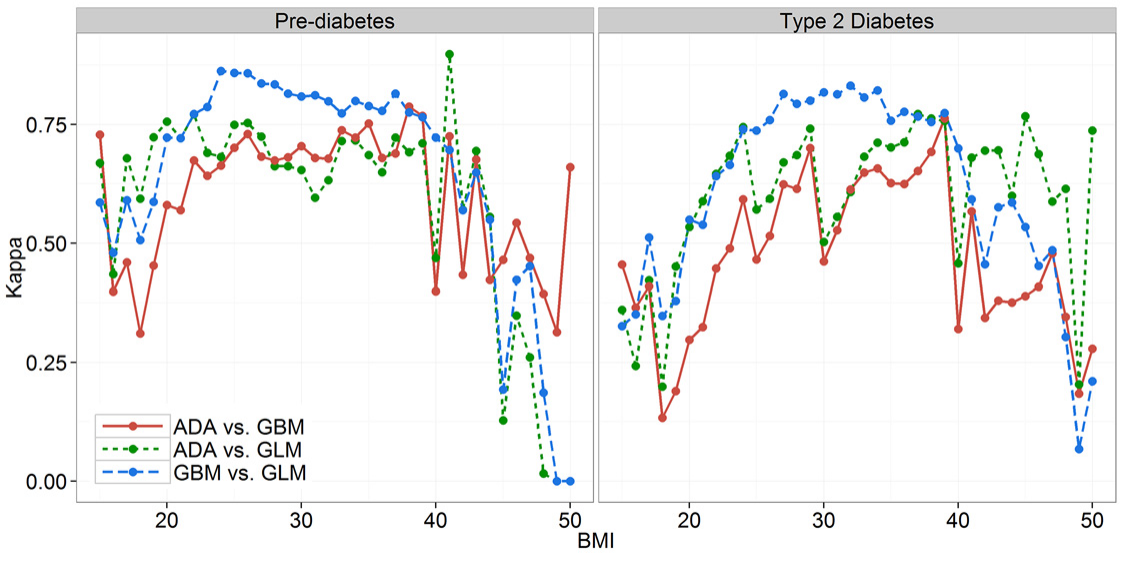
Agreement of predictive models by BMI for two classification problems.

Fig 3 compares Kappa coefficients calculated over 1000 holdout sets of 7103 randomly selected test cases for each pairwise comparison. In some regions the agreement levels do not differ among the three compared methods, but there are some regions (e.g. BMI range from 25 up to 35) with higher agreement levels for GLM and GBM. The sharp drops in agreement of ADA vs. GLM and ADA vs. GBM at BMI of 30 and 40, especially in case of diabetes prediction, are also noticeable.

**Fig 4:**
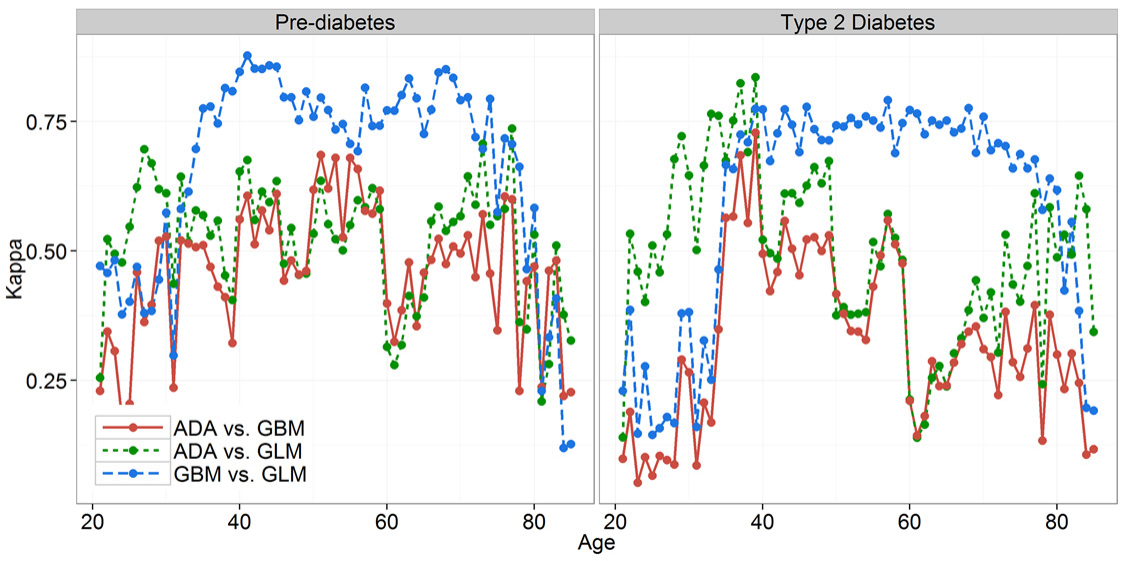
Agreement of predictive models by age for two classification problems.

Fig 4 presents similar results where Kappa is observed in relation to the age of persons. The discrete boundaries for age from the ADA questionnaire can be seen, especially for people aged 59 or over where Kappa drops significantly for comparisons with ADA, while GLM and GBM keep the agreement at a high level. It is also possible to observe lower levels of agreement even for GLM vs. GBM comparison, but only in extremely young or old population where sample sizes are small and consequently result in higher variances.

## Discussion and Conclusions

Predictive models play an important role in forecasting health related patient outcomes, which is increasingly recognized as an essential activity in clinical decision making, clinical research, and healthcare quality assessment [33]. A study by Buijsse et al. [34] systematically explored methodological issues in diabetes risk assessment tools and observed that most risk assessment tools demonstrated high performance in datasets and populations where they were developed. However, the performance dropped in external populations. In this respect, authors suggest that risk assessment tools should be validated in populations with the characteristics of the original population that was used for the development of such tools. Therefore, this study focuses on risk assessment tools from developed countries with similar characteristics of population. There are some limitations in characteristics such as race and ethnicity, where the distribution can vary among different countries. This could be one of the reasons for good performance of the ADA questionnaire that was specifically developed for the US population. The ADA model allowed better insight into the gap between using the paper-and-pencil questionnaire-based models and specific machine learning based approaches in online settings. An additional limitation in comparison of diabetes risk assessment models lies in the fact that some models aim to predict the risk of undiagnosed diabetes while other models aim to predict the onset of diabetes in the future (e.g. in five or ten years from now). This study partially solves this problem by setting the threshold level based on the training dataset performance.

One of the main reasons for weak performance of screening tools is related to the problem of discretization used with numerical values in paper-and-pencil based questionnaires. Observing the common set of variables used in this study, the numerical variables (age, BMI and waist circumference) were used much more efficiently by GLM and GBM in comparison to their interval-based representation in the ADA model. This can be demonstrated in the Kappa analysis section with an increase of disagreement between the ADA and the other two methods at the beginning and end of the age or BMI intervals. There is room for improvement of performance for GLM and GBM by replacing the binary variable for hypertension with average diastolic and systolic blood pressure measurement that are also available in NHANES data, but were not used in this study.

The screening of populations for risk of diabetes and pre-diabetes is an important step in reducing the burden of costs related to diabetes complications for policy makers [35]. Therefore it is important to select the appropriate number of candidates for further screening. The models compared in this study show that by optimizing for AUC does not necessarily result in an optimal number of selected persons. Only GLM and GBM came close to the percentage of selected persons for screening when comparing the results to a study by Bang et al. [8] evaluated on NHANES 2005–2006 data. The fact that in the present study there is a higher percentage of selected persons for ADA could lie in a different distribution of the data, especially in recent years with an increased prevalence of diabetes (Bang et al. report undiagnosed diabetes prevalence of 2.8% compared to 4.4% in this study).

Interestingly, a study by Robinson et al. [15] shows no significant differences in predictive performance when comparing the results obtained from electronic and paper based risk score. However, our study shows that there is a large gap in performance when comparing currently used paper based models to simple statistical methods such as GLM and GBM that are simple to implement in online settings. It is therefore very important to take great care and consider optimizing the online versions of questionnaires that were primarily developed as classical paper questionnaires.

It is possible to hypothesize that this gap does not exist only in online risk tests on websites, but also in a wide range of mobile applications where most applications use paper-and-pencil oriented models similar to the models described in this study [36, 37]. Further research using most frequent screening tools in mobile diabetes risk assessment applications would be needed to confirm this hypothesis.
